# The Use of the Gum Lancet

**Published:** 1883-03

**Authors:** 


### THE USE OF THE GUM LANCET.

The almost indiscriminate lancing of the gums of teething children is a practice that is, happily we believe, falling into desuetude. It has been the habit of many dentists, and physicians as well, to ascribe to teething most of the ills to which children are subject during that critical period of the change from an exclusively fluid to a more concentrated diet. It is needless to remind any intelligent practitioner that the digestive apparatus of the infant is unfit to receive anything but the very simplest aliment. Yet injudicious mothers too often give to their young children food that would try the stomachs of strong men, and the consequences are what might naturally be expected.

Children are born without teeth, because their as yet imperfect and weak digestive functions are not fitted for the reception of food which would require a masticatory apparatus. As they gain in strength and in digestive power the teeth begin to make their appearance ; the incisors or cutting teeth first, to indicate—not that the period for a change of nutriment has arrived—but that the time is approaching, and that nature is beginning to make provision for one of the great eras of a human life. It is not until the child is at least two and a half years old that the dental organs are sufficiently developed to properly prepare a more solid diet for digestion. Even at that time nothing but extremely succulent foods are permissible, and during the whole period of the dependence of the child upon the deciduous or temporary teeth it is unfitted for the regimen suitable to adult life. Nature gives an unfailing sign of the proper time for dietary advancement, not only in the natural appetites of the young of all mammals, but in the regular succession of their dental organs, and it is the extreme of folly to incite the appetites of children with temptingly prepared solid food, until both the teeth and the digestive apparatus are sufficiently developed to properly prepare and take care of it.

Mothers are usually, for divers reasons, in great haste to wean their children, and with the advent of their first tooth they begin to stuff them with vegetable, and even with animal food. The first attempts are usually made with viands that have been first masticated and mixed with the saliva of the mother—the saliva of an adult, that is as unfitted for the stomach of a nursing infant as is the food which it is used to moisten. Watch an infant thus fed and see the wry faces and grimaces with which it rebels at, and usually repels the unfit stuff. Tea, coffee, and other stimulants are administered, and after a period of rejection a morbid appetite is finally awakened, and before the appearance of the first molar tooth the child eagerly swallows sustenance which its stomach and intestines cannot digest, and which if assimilated at all is sure to bring on pain and disease. About the time when the molar teeth are in process of eruption digestive ills of all sorts attack the child, and to the mere physiological process of cutting of the teeth is attributed the flatulence, diarrhea, convulsions and death of the little innocent, which falls a victim to the ignorant and mistaken kindness of its mother.

The child is ill. There is fever, with all sorts of digestive disturbance. Within its immature stomach lies a mass of food which it is powerless to digest, and which the whole system is rebelling against. The nervous irritation is so intense that possibly convulsions ensue. The physician or dentist looks in the mouth, and, incapable of seeing further, attributes all the trouble to a tooth which is perhaps not within a month of its proper period of eruption. Out comes the knife or lancet and the tender gums are lacerated, nine times out of ten without the slightest benefit. The tenth time the irritation is, by a kind of metastasis, transferred to the mouth, and temporary relief perhaps obtained. But the true cause of the distress is not diagnosed, and a repetition of the murderous feeding finally sweeps the little sufferer out of the reach of foolish mothers and meddling practitioners.

The assertion has been made that more than half the human family die of diseases incident to teething. A more false and irrational statement could not well be put forward, if by this is meant that these disorders are induced by the cutting of the teeth. Whoever heard of a child that suffered from the cutting of its teeth, which had a full supply of mother's milk during that period, and which was allowed no other food? Is not such an one almost universally a sturdy brat, that knows not what stomach-ache is like? On the other hand, is it not the truth that the infant which is deprived of its natural food, either through the criminal unwillingness of the mother to bear the proper burthens of maternity, or her impotent incompetence to properly nourish the offspring which she has brought into the world, is frequently a weak, puny, scrawny child, which, ten to one, falls a victim to its improper food before the time arrives when its organs were fit for that which has killed it?

Inflamed and tumid gums are too often the result of an irritated and angry stomach. Digestion and assimilation being properly performed, there is nothing in the mere process of the eruption of the teeth which can cause any serious disturbance. Nature has provided for the absorption and disappearance of the tissues covering the growing tooth without any febrile symptoms, any diarrhœa or nervous convulsions, and the dentist or physician who mistakes digestive and functional disturbances for a mere local irritation would do better to go back to his college and his books and commence a new course of study. The mere fact that the period for the manifestation of these symptoms of a vicious diet is nearly coincident with that of cutting the molar teeth ought not to mislead any one.

There is usually little trouble during the eruption of the incisor teeth, unless the food has been exceptionally bad from the first, for the injudicious feeding does not commonly begin until near this period. Between the time of the appearance of the central incisors and that of the second molars there is plenty of opportunity for ruining the digestion of the little ene. It is the cutting of these last that is dreaded by fond parents. So universally is their eruption coupled with digestive disorders that they are popularly known by the name of "stomach-teeth." Yet there are no greater obstacles in the way of their proper succession than in those of any of the others. The gums and investing tissues have not such an exquisitely nervous organization, are not so thoroughly supplied with nerve fibres as to produce any grave disturbances by the mere force of an advancing tooth. There is little or no sensitiveness in the raised gum. Pressure over the coming tooth is not annoying to the infant. On the contrary it often seems grateful. Whence then this idea of dangerous reflex nervous disturbances? The tissue is not highly vascular and there is consequently no serious interference with the circulation. What excuse is there then, except in unusual cases, for lancing the gums of the teething child?

We do not mean to assert that the interference of the practitioner is never demanded, but we do firmly believe that even now, when the gum lancet is resorted to so much less often than formerly, there is no call for its use once where it is employed ten times.

